# A qualitative inquiry of experiences of HIV-related stigma and its effects among people living with HIV on treatment in rural Kilifi, Kenya

**DOI:** 10.3389/fpubh.2023.1188446

**Published:** 2023-06-21

**Authors:** Stanley W. Wanjala, Moses K. Nyongesa, Rachael Mapenzi, Stanley Luchters, Amina Abubakar

**Affiliations:** ^1^Department of Public Health and Primary Care, Faculty of Medicine and Health Sciences, Ghent University, Ghent, Belgium; ^2^Department of Social Sciences, School of Humanities and Social Sciences, Pwani University, Kilifi, Kenya; ^3^Neuroassessment Group, KEMRI/Wellcome Trust Research Programme, Centre for Geographic Medicine Research (Coast), Kilifi, Kenya; ^4^Department of Clinical, Neuro- and Developmental Psychology, Vrije Universiteit Amsterdam, Amsterdam, Netherlands; ^5^Institute for Human Development, Aga Khan University, Nairobi, Kenya; ^6^Centre for Sexual Health and HIV AIDS Research (CeSHHAR), Harare, Zimbabwe; ^7^Liverpool School of Tropical Medicine, Liverpool, United Kingdom; ^8^Department of Public Health, School of Human and Health Sciences, Pwani University, Kilifi, Kenya; ^9^Department of Psychiatry, University of Oxford, Oxford, United Kingdom

**Keywords:** HIV-infection, adults’, people living with HIV/AIDS, in-depth interview(s), qualitative inquiry, Kenya, HIV-related stigma

## Abstract

**Background:**

The pervasiveness of HIV-related stigma and discrimination, and its consequences on HIV prevention and treatment, have been well documented. However, little is known about the lived experiences of HIV-related stigma and its effects among the general adult population living with HIV in rural African settings. This study set out to explore this knowledge gap.

**Methods:**

From April to June 2018, we conducted in-depth interviews with a convenience sample of 40 adults living with HIV aged 18–58 years in Kilifi, Kenya. A semi-structured interview guide was used to explore experiences of HIV-related stigma and its impact on these adults. A framework approach was used to analyze the data using NVIVO 11 software.

**Results:**

Participants reported experiences of HIV-related stigma in its various forms (anticipated, perceived, internalised, and enacted), as well as its effects on HIV treatment and social and personal spheres. The internalisation of stigma caused by enacted stigma impacted care-seeking behavior resulting in worse overall health. Anxiety and depression characterized by suicidal ideation were the results of internalised stigma. Anticipated stigma prompted HIV medication concealment, care-seeking in remote healthcare facilities, and care avoidance. Fewer social interactions and marital conflicts resulted from perceived stigma. Overall, HIV-related stigma resulted in partial and non-disclosure of HIV seropositivity and medication non-adherence. At a personal level, mental health issues and diminished sexual or marital prospects (for the unmarried) were reported.

**Conclusion:**

Despite high awareness of HIV and AIDS among the general population in Kenya, adults living with HIV in rural Kilifi still experience different forms of HIV-related stigma (including self-stigma) that result in a raft of social, personal, and HIV-treatment-related consequences. Our findings underscore the urgent need to reevaluate and adopt more effective strategies for implementing HIV-related anti-stigma programs at the community level. Addressing individual-level stigma will require the design of targeted interventions. To improve the lives of adults living with HIV in Kilifi, the effects of HIV-related stigma, particularly on HIV treatment, must be addressed.

## 1. Introduction

Stigma is one of the major social determinants of health that has been described as a ‘hidden’ burden of disease (1). Stigma can be reflected in experiences, including enacted or experienced stigma, and in attitudes either conceptualized as anticipated, internalised, or perceived stigma that affects a particular trait or mark (2, 3). Enacted or experienced stigma refers to discriminatory acts or behaviours, such as prejudice and/or discrimination from others because of one’s HIV infection status (2, 4). Anticipated stigma depicts the expectations of stigma experiences (enacted stigma) happening in the future (2). Internalised stigma alludes to an individual’s awareness, acceptance, and application of stigma to oneself (5, 6). Lastly, perceived stigma refers to a person’s understanding of how others may act towards, feel or think about an individual with a particular trait, attribute, or identity (7).

HIV-related stigma and discrimination or the devaluation and rejection associated with an individual’s HIV diagnosis is a human rights violation (8, 9). It has proven to be a persistent barrier to HIV prevention, control, and treatment (10, 11) by delaying HIV testing (12) and engagement with care (13). In addition, HIV-related stigma and discrimination have been linked to negative impacts on various aspects of the lives of people living with HIV/AIDS (PLWHA), including, disclosure (14), antiretroviral therapy (ART) adherence (15), mental health, and interpersonal relationships (16). Studies have shown that despite the expanded access to ART, the fear of stigmatisation has resulted in suboptimal adherence to treatment among PLWHA (17), leading to virological non-suppression (18, 19), increased disease progression (20), a higher risk of HIV transmission (21) and death (22). Furthermore, HIV-related stigma has been linked with negative outcomes such as non-disclosure of HIV-positive status with PLWHA hesitant to reveal their HIV status to others due to fear of discrimination and social isolation (16, 23). This fear of disclosure and social isolation leads to poor mental health functioning including anxiety and depression (16, 24), which may further impede progress toward HIV eradication. Furthermore, the fear of discrimination and public ridicule due to HIV status disclosure has been shown to negatively impact the interpersonal relationships of PLWHA by disrupting their everyday social relationships/interactions resulting in a loss of social support, isolation, and loneliness through social disengagement and an unwillingness to participate in social activities (16, 25). The disruption of their normal social relationships especially in marriage makes it difficult for them to associate with others including finding a partner (16).

While much attention has been focused on stigma, even becoming the focus of the World AIDS campaigns for 2002–2003, HIV-related stigma persists in sub-Saharan Africa (SSA) (26, 27), with deleterious consequences on both the infected and affected. Despite there being quantitative literature on the impacts of HIV-related stigma among adults living with HIV, qualitative data on this subject is insufficient, especially in SSA. Existing qualitative studies on this topic in SSA mainly report the views of adolescents and young adults (28–33), including adolescent girls and young women (34). Some qualitative and quantitative knowledge about HIV-related stigma is available in Kenya. However, these studies only focus on the experiences of adults living with HIV (35, 36), including specific groups of women (36), or explore the potential of livelihood intervention to reduce the stigma of HIV (37). This qualitative study will assist us in gaining a contextualized, rich, detailed, and deeper understanding of the lived experiences of HIV-related stigma and its effects on this particular population. Lived experiences are an individuals’ subjective experiences, perceptions and emotions of people affected by HIV stigma which can include a wide range of outcomes such as, treatment outcomes, adherence, disclosure, mental health outcomes and socialization. To the best of our knowledge, only one study has gone beyond exploring lived experiences to report the impacts of HIV-related stigma on ART adherence among adults (38), but not any other cascade of effects that HIV-related stigma portends for this population. To address this knowledge gap, our study aimed to:

a) Explore the lived experiences of HIV-related stigma among adults living with HIV in a rural Kenyan setting – Kilifi; and
b) Understand the effects of HIV-related stigma on disclosure, ART adherence, mental health, and the social interactions of these adults.

## 2. Methods

We describe study methods and findings in accordance with the COREQ (Consolidated Criteria for Reporting Qualitative Research) checklist (39) (see Supplementary Table S3). This checklist includes 32 items that must must be addressed in order to report qualitative studies explicitly and comprehensively (39).

### 2.1. Study site

This study was conducted in Kilifi County – coastal Kenya, through the Centre for Geographic Medicine Research-Coast (CGMR-C), Kenya Medical Research Institute -Wellcome Trust Research Programme (KEMRI/WTRP), between April and June 2018. Kilifi County is one of the six counties in Kenya’s Coastal region and among the poorest counties in Kenya (40). Literacy levels in this county are low, with high rates of school dropouts (41). In 2017, an estimated 30,597 adults (15–49 years old) were living with HIV in Kilifi county, representing an estimated HIV prevalence of 3.8% (male = 2.3%, female = 5.4%) (42).

### 2.2. Study participants, sampling, and recruitment procedure

Participants were 40 adults aged 18–58 years old, living with HIV and attending an HIV care and treatment clinic at the Kilifi County Hospital: a teaching and referral hospital. These adults were identified using records from a larger research project conducted at this health facility aimed at understanding important outcomes in adults living with HIV like health-related quality of life, HIV-related stigma, and mental health outcomes. The larger study recruited a total of 450 (79.1% female and 20.9% male) adults 18 to 60 years, with confirmed HIV-positive status and on antiretroviral treatment from the HIV clinic at the Kilifi County Hospital (43, 44). Participants were conveniently selected depending on their availability and willingness to participate following an initial phone call by a research team member. Participants were recruited from the list of potential participants balancing the distribution by age, gender, and geographic location. The emergence of data saturation (45, 46) determined the sample size as new interviews yielded little or no new information or insights following debriefing sessions by interviewers during the data collection exercise.

### 2.3. Data collection procedures

Data was collected through face-to-face in-depth interviews with individual participants lasting 30–45 min. A semi-structured interview schedule (see Supplementary Table S1) with specific probe lines for the main stem question was used to guard, albeit not completely, against interviewers drifting off the topics under inquiry. The interview schedule covered topics around participants’ experience of HIV-related stigma and its direct impacts on their treatment outcomes and other aspects of their life, e.g., personal and social spheres. Additionally, all interviewers were trained around the interview process, including what to ask and how to probe (with rehearsal sessions). Debrief sessions between the interviewers and the larger research team were held daily to discuss interview experiences including probing challenges. Interviewers compared and contrasted collected data in order to identify emerging patterns, themes and commonalities. Interviews took place in a quiet private room at the HIV care and treatment clinic, Kilifi County Hospital. The interviews were conducted by researchers representing three distinct specializations: (1) A mental health expert with global health training and expertise (M.K.N.), (2) a medical sociologist with expertise in HIV/AIDS stigma (S.W.W), and (3) a trained fieldwork assessor (R.M.) (see Supplementary Table S2). All interviews were conducted in Kiswahili (the official national language of Kenya) and recorded digitally. All three interviewers were Kenyan nationals, fluent in Kiswahili.

### 2.4. Data management and analysis

Data were managed and analysed using Nvivo software (version 11 Pro, QSR international). Audio-recorded interviews were transcribed verbatim by our experienced team of transcriptionists at the department, who are also fluent in Kiswahili, removing all personal or identifying information, and then translated into English by experienced translators in the research team. The authors selected interesting quotes, reviewed the English translations for accuracy and uploaded them on Nvivo. Data analysis was conducted by authors (SWW and AA) in the original language – Kiswahili using the framework approach as described by Ritchie and Spice (47). The analysis included: i) immersion into the data, i.e., reading and re-reading the transcripts, ii) creating an initial coding scheme (by SWW and AA). Codes were inductively generated (through an in-depth reading of the transcripts) and deductively (by drawing on questions from the interview guide), iii) Coding – the coding process was continuously expanded to incorporate emerging themes, and iv) SWW and AA generated a comparative analysis of codes. Authors SWW and AA familiarized themselves with five randomly selected transcripts and independently coded them. Any differences arising from the coding were resolved through mutual agreement. SWW and AA then held a meeting after individually coding three more transcripts. SWW coded the remaining transcripts because the coding pattern was nearly identical. In the case of emerging codes outside the agreed-upon coding framework, SWW consulted AA before updating the codebook. After coding all the data in Nvivo, related themes were categorized and exported to Microsoft Excel to generate charts/matrices. Finally, charts were used to summarize data, identify differences or similarities, and explore patterns in the analysed data. Ethical considerations.

Ethical approval was granted by the local institutional review board, Scientific and Ethics Review Unit (SERU; Ref KEMRI/SERU/CGMR-C/108/3594). Besides, authorization to work in the HIV care and treatment clinic was obtained from the Ministry of Health, County government of Kilifi (Ref HP/KCHS/VOL.VIX/65). Potential participants were acquainted with the study objectives and their right to decline participation in the study outright or withdraw consent at any research stage without any undesirable consequence. All participants provided written, informed consent to be part of the study.

## 3. Results

### 3.1. Socio-demographic characteristics of respondents

Table 1 presents the socio-demographic characteristics of the 40 interviewed participants which comprised 50% males and 50% females. The mean age of participants was 30.5 years (range 18–58). Twenty-one participants had a secondary or tertiary level of education, with 33 Christians and 6 Muslims among them.

**Table 1:** Summary of participants’ socio-demographic characteristics.

Sample characteristics	Frequency (%)
Socio-demographic characteristics	
Age	
Range	18–58 years
18–24 years	22 (55%)
25–58 years	18 (45%)
Sex	
Female	20 (50%)
Male	20 (50%)
Level of education, OM = 1	
None	1 (3%)
Primary school	17 (44%)
Secondary school	15 (38%)
Tertiary	6 (15%)
Religion, OM = 1	
Christian	33 (85%)
Muslim	6 (15%)

OM = Observation with missing value.

### 3.2. Participants’ experiences of HIV-related stigma and stigma effects on HIV prevention and treatment

**Figure 1:**
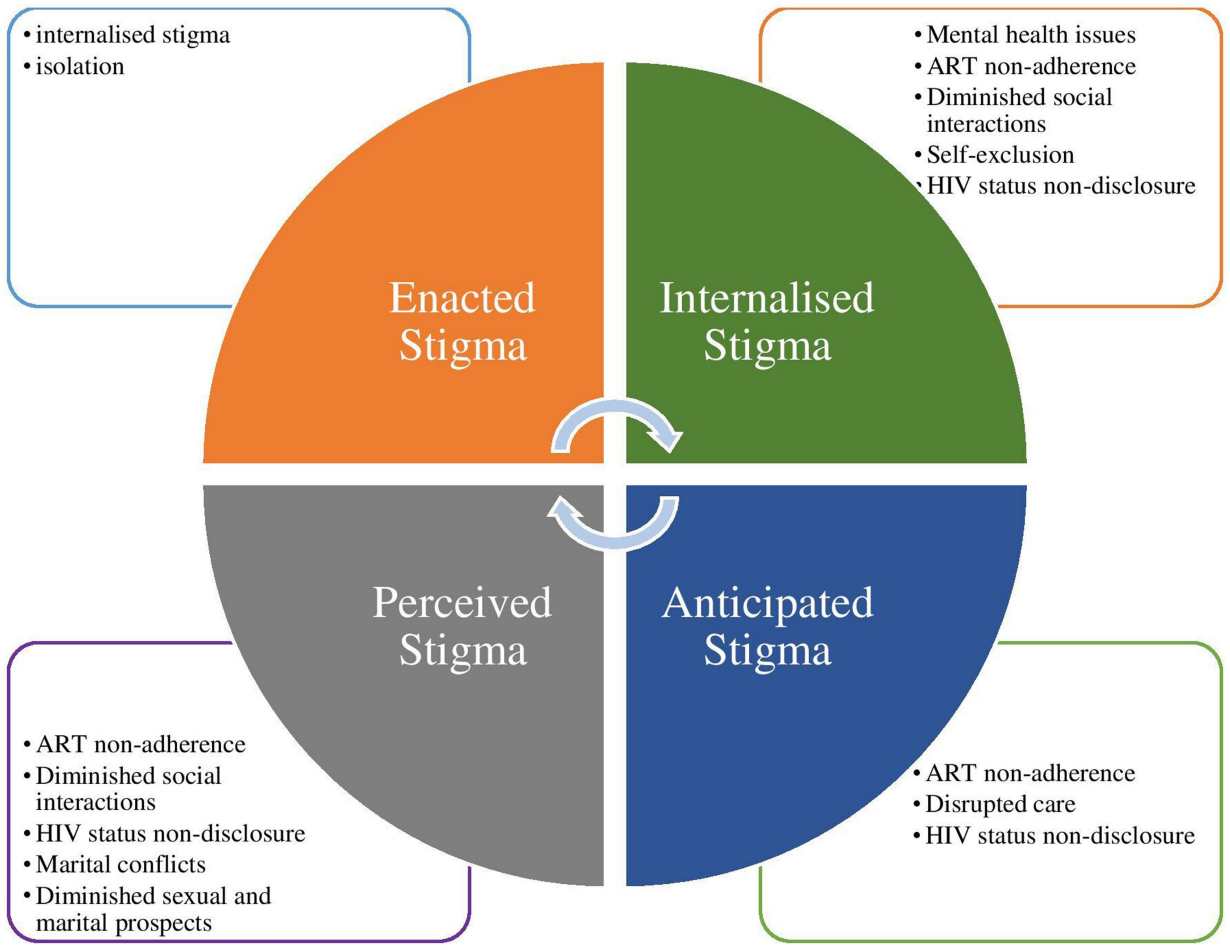
Participants’ experience of HIV-related stigma and stigma effects on HIV prevention and treatment.

Participants described their experiences with various forms of HIV-related stigma in our context. These include enacted, internalised, anticipated, and perceived stigma. These forms of stigma had varying effects on different spheres of life of the participants, as summarized in Figure 1.

#### 3.2.1 Experiences of enacted HIV stigma.

Participants shared their personal accounts with HIV-related stigma, which was manifested through various forms of verbal discrimination, such as blaming, humiliation, rejection, and social exclusion, which stemmed from the fear of contracting the virus or interacting with PLWHA. They presented concrete instances of how people with HIV face such stigma. Moreover, the participants elaborated on how their loved ones and community members habitually resorted to name-calling and labeling to demean and belittle PLWHA. Some of the examples included referring to them as cursed, already dead, or close to death, as demonstrated by the following quotes:

*At times, individuals who are aware of your HIV status may engage in gossip about you. They may deliberately speak in a loud or exaggerated manner so that you can hear their words, which can lead to feelings of humiliation. (55-year-old-female)“[……….] Someone will tell you that that family has been cursed, they have the ‘disease of the cursed,’ they are worthless, and nobody is supposed to be close to them” (21-year-old-female)*

*“Aaaaah! You are aware that XXXX’s mother is infected. If you become friends, do not extend your hand to her when you greet her. If you do, you will catch those germs (HIV) as well. If they request for water, dispose of the cup in the toilet or break it when they are done.” (55-year- old female)*

One respondent expressed frustration at how PLWHA are treated by family members, while acknowledging that living with HIV can be challenging but does not preclude one from studying, working, or leading a fulfilling life. He also stated that PLWHA can live productive lives just like anyone else, but require support and understanding from others in the community to do so.

*“If you live with a stepmother or an aunt who knows you are infected, they regard you as nothing and a dead person. We (PLWHA) are referred to as moving corpses, but this is false. We can work, like other people, study, be engineers, and be teachers, but where do we get that support from?” (22-year-old male)*

Respondents described how they were ostracized from social activities and how physical contact with them was avoided, especially by HIV-negative family members. This was due to misinformation and the belief that HIV could be transmitted through casual contact.

*During mealtime, either people are warned against sitting close to me, or I am instructed to go and eat from outside. I have my own plates and cups, and I am not allowed to use any utensils apart from what is reserved for me. This makes one’s health deteriorate due to too much rumination/thinking too much. (32-year-old-female)*

*“…when I used to visit my aunt, my utensils were kept separate from the rest of the family, and I was not allowed to watch television with them because they said I would infect them” (19-year-old-male)*

Other forms of exclusion in the community included denial of employment and educational opportunities as a result of one’s HIV status, which resulted in a loss of economic security as PLWHA struggled to find work:

“*An opportunity to join the police force presented itself, but I was instructed not to attend the recruitment exercise because of my HIV status. I believe I would be working if I had gone.*” *(24-year-old male)*

*A parent might decide to withdraw a young person living with HIV from school so that his/her sibling who is not infected can continue with his/her education. The parent will say, “let me not educate this one (HIV-infected youth) because I know I am educating him/her, and he/she might die any day. It is better to educate the uninfected so that they can help me later in life than to educate this one (infected) who will die and will not assist me.” (19-year-old male)*

Discrimination was also evident in the school setting, owing to inadvertent disclosure by classmates. Because they were HIV positive, their classmates declined gifts, and their deskmates avoided contact by not sitting next to them.

*[………] students are often problematic and stubborn at school. If they know about your HIV status, your deskmate can run away from you when he/she is told “you are sitting next to an HIV-infected person [……………]. ‘I cannot sit next to an HIV-infected person.” Sometimes during break time, you might have bought stuff to share with your friends; when your colleagues are offered by their friends, they accept, but when you want to share yours with them, they decline the offer. “He/she is HIV-positive, do not accept or eat whatever they are offering” (20-year-old female)*

#### 3.2.2. Experiences and effects of internalised HIV stigma

Participants admitted that they had internalised fears, social attitudes, and self-discriminatory behaviours at some point in their lives. They reported psychological difficulties such as low self-esteem, sadness, mood swings, anxiety, and depression, often leading to thoughts of suicide. They found it hard to accept their HIV status, making it difficult for them to disclose their status, adhere to their antiretrovirals, and engage with others in the community. Due to internalised stigma, they experienced self-loathing, hopelessness, and a lack of self-confidence. Additionally, they described feeling unworthy, which negatively affected their relationships with loved ones.

*“It makes one lose confidence in themselves [………] It also leads to living in denial and self-hate, making them often ruminate. They question why they were born and why they are how they are (with an HIV positive status)” (19-year-old male)*

*Due to discrimination based on your HIV status, you may question why you are being treated unfairly, feel self-hatred for not knowing your actions could lead to contracting HIV, and experience rumination as those who know your status discriminate against you, ultimately leading to thoughts of suicide as a way to end the negative experience (46-year-old-male)*

*Sadness is a result of discrimination. You question many things. “Had I not had this disease, they would not be discriminating against me. Had I not been born with this disease, they would not be discriminating against me. So you become sad and isolate yourself.” (24-year-old female)*

Internalised stigma has also been linked to psychological distress, which can negatively impact a person’s overall well-being, leaving PLWHA confused and unsure of why they were taking their ARVs, leading to ART non-adherence:

*[………….] mental health translates to physical health. So once you have been psychologically affected, your life is no longer complete. Now you will find that you will start experiencing mood disturbances, and sometimes you become confused as to even why you are swallowing these drugs (ARVs), and you will also notice that your health deteriorates. (44-year-old male)*

*If he experiences discrimination, he may feel ashamed, embarrassed, and question the purpose of his existence, leading to a sense of not being okay, which could potentially result in him not adhering to his medication (54-year-old-male)*

Participants described how PLWHA internalizing stigma by repeatedly focusing on negative thoughts and experiences could cause additional distress and potential harm. Individuals may feel overwhelmed by their negative emotions as a result of their feelings of discrimination and isolation, making them more prone to suicidal ideation as a way out of their suffering and relieving the burden on their families:

*…… Sometimes you feel like people have discriminated against you and do not want to associate with you. You are left alone and will start questioning why you adhere to your medication. You always think you are unwanted, and this rumination might lead to suicide.” (45-year-old-female)*

*“[……] Someone might be disturbed. They will think about so many issues. They will even contemplate committing suicide by drowning themselves in the ocean as a result of the stress they are undergoing.” (22-year-old-male)*

Prior negative stigma and discrimination experiences prevented PLWHA from socialising and associating with others in their social circle. They were also excluded from potentially beneficial support groups. This self-exclusion made it difficult for PLWHA to connect with others who had similar experiences and could offer emotional support, guidance, and practical help.

*“[…….] if you have had prior stigmatisation, you fear associating with others because you think they will stigmatize you. This makes one exclude themselves from support groups that are very helpful” [22-year-old-male]*

A participant recounted a personal experience she had when she was in primary school. She had not disclosed her HIV status to her classmates at school, but whenever they discussed HIV, they would stare at her because of her physical appearance (skinny), making her uncomfortable and self-conscious, and she eventually began to isolate herself from her peers and spend time alone.

*“I remember when I was a class five student in primary school, I had not disclosed my HIV status to my peers. Due to being skinny, anytime my peers started discussing HIV, others would stare at me because of how skinny I was, and it reached a point I isolated myself from others and started spending time alone” (24-year-old-female)*

Furthermore, dealing with a personal and chronic health condition such as HIV can made it difficult for one to concentrate on studies as the resulting thoughts and concerns were a distraction, making it challenging for one to pay attention to what was being taught in class. Internalised stigma affected learners’ concentration at school, impeding their learning experience, as illustrated by the following quote:

*“[………] as a young person in school, instead of concentrating in class and listening to what the teacher is saying, you will always be engulfed in thoughts thinking about how to cope with your situation (HIV status), thus not learning as expected” (20-year old female)*

A participant reported having conflicting feelings about how to deal with forming social relationships while concealing their HIV status and avoiding the spread of the HIV virus:

*[…….] At my age, someone would love to be in a relationship with a boy; if he is a boy, he would want to be with a girl. So he/she will ask himself/herself so many questions. I am HIV positive? I am in love with [boyfriend]. How do I disclose to [boyfriend] that I am HIV positive? I also do not want to infect [boyfriend] because it is not good to infect him/her. What if he/she sees my drugs because I carry them daily as we go out, and I take them (drugs) at appointed times? He/she might ask me. So, it is better not to disclose it and stop taking medication. […………] you will be scared, you will have fear if you disclose to him/her, he/she will leave you, and you are also scared of spreading the virus. (21-year-old female)*

#### 3.2.3. Experiences and effects of anticipated HIV stigma

Fewer participants expressed concerns about anticipated stigma, including the fear of negative attitudes and actions towards PLWHA, such as gossip, loss of relationships, and lack of social support. This fear of stigma acted as a hindrance to engaging in care, leading to behaviours such as avoiding clinic visits to avoid being seen, partial or complete non-disclosure of HIV status, hiding medication, and seeking treatment at distant facilities. Moreover, some participants refrained from disclosing their HIV status to family and close friends to avoid losing social support. Participants believed non-disclosure was the best way to avoid the expected negative social consequences, such as gossip and humiliation.

“*Yeah, disclosing your status is okay, but you cannot disclose it to everyone. This is because you can decide to disclose to a certain individual and not know what that person is like. So you will reveal it to them, and then they go and disclos; thus, your status (HIV) becomes known throughout the village. Once you get to hear about this, you will not be happy. (20-year-old female)*

*“[……] HIV status disclosure is good, but it is complicated for you to disclose because of the fear of gossip, especially if you have experienced stigma” (22-year-old-male)*

*“You can only disclose to someone who can keep your secret (HIV status)” [54-year-old male]*

A participant reported having concerns about disclosing her HIV status to her children or family, as this could affect the parent–child emotional bond making it difficult for her to receive the social support and care she needs.

*“I am infected; how will I live? I do not want my children to know about my HIV status because they might not be willing to care for me. I will not disclose to my children or family” [55-year-old-female]*

A participant reported struggling with the difficult decision of whether or not to disclose her HIV status to her employer, citing the complex and sensitive nature of the issue that requires careful consideration of the potential risks and benefits of disclosure. She is concerned about potential discrimination or losing her job and is unsure whether her employer will still accept her after she discloses her HIV status:

*It is not an easy thing to disclose. I have disclosed my status to some people but not my employer because I still want to live with them so that whatever little they pay me helps me to get along. If I tell them the truth about my HIV status, will they still accept (employ) me? (32-year-old female)*

##### 3.2.3.1. Anticipated stigma management or coping strategies

The apprehension of facing stigma impacted participants’ willingness to disclose their HIV status, seek care, and adhere to antiretroviral therapy (ART). To prevent inadvertently revealing their seropositive status while seeking HIV care, participants adopted coping mechanisms like seeking treatment at faraway locations, declining clinic-offered incentives, avoiding services exclusively for PLWHA, or avoiding care altogether. One participant mentioned feeling embarrassed and, hesitant to disclose their HIV status.

*“[…………] who wants to be embarrassed or slandered in front of people? No one. So you will have to keep your status to yourself (secret).” (31-year-old female)*

Living with HIV can be a sensitive and personal matter, and it often carries a lot of social stigma and discrimination. According to a participant, PLWHA are skeptical about disclosing their HIV status to people who are not HIV-positive for fear of being judged, rejected, or discriminated. They are only free to disclose their HIV status to other PLWHA because they are similar to them and would understand them better:

*“Unless both individuals are living with HIV, they will not be comfortable disclosing their status and even advising each other because of the fear that they harbor inside them” [31-year-old-female]*

According to one participant, some PLWHA started refusing clinic-offered incentives such as mosquito nets and water jerry cans because they did not want to draw attention to themselves as HIV-positive. This exemplifies how stigma can affect the behavior and choices of PLWHA:

*“……. We used to be given water jerry cans, mosquito nets, and other items at the clinic. To protect themselves from stigmatisation, people started refusing the incentives as they acted as identifiers for PLWHA” [31-year-old female]*

A patient expressed feelings of embarrassment or shame at being seen taking her ARVs or attending an HIV clinic. As a strategy to avoid anticipated stigma, she hides her medication and takes it in secret. This can result in her missing doses or not taking her medication at the correct time, which can negatively affect her health.

*“The first thing is you will not want to be seen with ARVs; you can even dig a hole to hide the ARVs and challenge people to come and search your house for ARVs. You will not want to be seen taking ARVs or attending the clinic. This will make you not take your ARVs at the appointed time because you will have to wait for people to leave so that you can ingest them” [22-year-old-female]*

Due to fear of stigma, some PLWHA were afraid of second-hand disclosure because they wanted to keep their status a secret from others. They felt more comfortable traveling long distances to get medication refills, in order to avoid the risk of being seen at their local clinic and having their status revealed. Others opted to hide while ingesting their ARV’s for fear of inadvertent disclosure.

*Interviewer: What will make this person not disclose their status?*

*Participant: “because they are afraid of second-hand disclosure. They want their status to remain a secret. That is why they might travel from fumbini to Malindi (70 kilometers away) to get a refill of their drugs”*. *[32-year-old-female]*

*If one is in a boarding school, it gets to a point where he/she finds it difficult to use their medication (ARVs); this forces them to hide when ingesting their ARVs so that others do not get to know about their status [21-year-old-female]*

A participant describes avoiding being near designated PLWHA areas (rooms and benches) at the healthcare facility because doing so could result in the inadvertent disclosure of their serostatus.

*“…. if I have been stigmatized at home, when I visit the clinic, I will not be comfortable sitting at the clinic’s waiting bay and will opt to go sit where other uninfected patients are so that people cannot identify me as having been infected with HIV” (22-year-old male)*

Respondents revealed that if a person believed that taking medication made them stand out or drew attention to their HIV status, they avoided taking their medication to blend in or fit in with the group and pass as healthy or normal like other HIV-negative members of society. They fear being identified as taking ARVs. As one participant stated:

*“If you experience discrimination because you are on ARVs, you will start questioning why you keep taking your medication. You will contemplate not taking your medication to be like your peers. Ultimately, you will stop taking your medication to pass as normal (HIV negative) as your peers” [19-year-old male]*

#### 3.2.4. Experiences and effects of perceived HIV stigma

Finally, participants recounted experiences where they or their children were stigmatized, revealing their apprehension about HIV-related stigma. Family members, friends, and community members were the most common perpetrators of this perceived stigma. For instance, one participant explained how parents would prevent their children from socialising with HIV-infected peers in their neighborhood due to a lack of understanding and fear of HIV transmission. Additionally, some participants shared that their friends were cautioned against touching them to avoid contracting the virus. These participants’ fears of discrimination due to their HIV status included exclusion from social activities, being unable to share food, isolation, and a spouse’s refusal to share the same bed after diagnosis. They also worried about the potential consequences of their HIV status being exposed publicly due to gossip and third-party disclosure. These anxieties were reported as barriers to some participants disclosing their HIV status and adhering to their medication regimen. Participants also expressed concern about missing job opportunities, leading to a loss of income and the ability to contribute to society.

*“Aaaaah! You know the mother to XXXX is infected. If you become friends, when you greet her, do not extend your hand to her. If you do, you will also get those germs (HIV).” (55 year- old female)*

*[………] He/she (HIV-negative person) can say this person is HIV-positive, so if he/she mingles with my children, [……] he/she may even infect them. You will realize that in this, he/she lacks understanding [21-year-old-female]*

Participant narratives revealed their concerns about being excluded from what others were planning, having fewer social interactions, and losing friends. The following quotes are illustrative:

*Incidences like people not wanting to be close to you. They plan things on their own, and you only come to learn about them later without your involvement whatsoever. (43-year-old male)*

*“You will have few friends. You might meet a friend who has accepted you as you are while others discriminate against you, hampering your social life*” *(36-year-old-male)*

*“[…] your friends will not want to associate with you, so you will find yourself lonely and sad because your friends hate you or dislike you.” (19-year-old-male)*

Participant fears also centered on marital conflicts for the married and loneliness, compromised intimate relationships, and diminished sexual and marital prospects for the unmarried, robbing them of the chance to live a normal adult life in which they could have their own families and a social support system. In addition, some participants do not disclose their seropositive status. Marital conflicts arose post-diagnosis, after their partners found out about their seropositive status:

*“For example, during a VCT visit, your wife’s HIV test could be negative while yours is positive. The counselor will provide post-test counseling and offer guidance on leading a healthy lifestyle. However, upon returning home, a conflict arises because your spouse will discriminate against you by not allowing you to sleep on the same bed. […….]. She chooses to sleep on her own bed, while you are left to sleep alone on another bed.” (43-year-old-male)*

*“The way people say, ahhhh! Young man, you need to marry. People do not know I have this illness (HIV). I keep quiet. Then I go sit down and ruminate. I am engulfed in thoughts. I say, Aaah! These people tell me to marry, yet I am the way I am (living with HIV). Most people are unaware of my serostatus. It [HIV status] is a secret known to my mother and me.” (24-year-old-male)*

A participant opined that if a parent is unable to meet the needs of their children, the children may begin to resent or dislike them. Children may feel neglected, frustrated, or angry if the parent is no longer able to meet their basic needs due to financial or other reasons as they used to do before their HIV diagnosis:

*“[…] your children might detest you because you can no longer provide for their needs as you used to.” [44-year-old-male]*

Participants’ narratives also highlighted HIV-negative individuals in society not wanting to come into contact with PLWHA by not wanting to touch their clothes.

*You see, you might have to hang your washing on the clothesline to dry, but someone pushes them aside so that they can hang theirs in a manner that they are not near yours (clothes). These are ways that can indicate that they are doing that because of your status (HIV). You will feel it, but you cannot say anything. [43-year-old male]*

Participant narratives also revealed fears of missing employment opportunities, thus losing out on means of earning a living and being productive members of society.

*For example, if it is an employment opportunity, someone will look at you and say, “Aaah! Because of his/her condition (HIV), they cannot perform well.” [19-year-old male]*

## 4. Discussion

### 4.1. Summary of key findings

Four decades into the HIV epidemic, HIV-related stigma is still of significant concern and has detrimental effects on the lives of PLWHA. This qualitative inquiry explored the experiences and effects of HIV-related stigma on the lives of adults living with HIV in a rural setting in coastal Kenya. Despite numerous efforts to eradicate HIV-related stigma, negative beliefs, the fear of contagion, and misconceptions about HIV are still prevalent in this setting, as described by participants’ diverse experiences. Our findings concur with a study in Vietnam (48), which demonstrated that the fear of HIV infection causes individuals to avoid contact with infected persons. The negative beliefs and misperceptions about HIV were partly exacerbated by ignorance and misinformation about the causes of HIV transmission. Notably, associating PLWHA with being cursed stigmatizes them as “*unwanted people*” in society. Furthermore, participants discussed the impacts of HIV-related stigma on HIV prevention and care because of its negative impacts on disclosure of HIV status, medication adherence, mental health, and social relationships, all affecting their economic and social support networks.

We discovered that understanding HIV-related stigma manifested as enacted, internalised, anticipated, and perceived stigma, an approach that has been applied in other settings (2, 49–51), was useful for us in analyzing and explaining HIV-related stigma and its manifestation among individuals living with HIV in our context (32, 36). Our study provides qualitative evidence showing that inadvertent HIV status disclosure is associated with enacted, anticipated, and perceived stigma. Evidence suggests that inadvertently disclosing one’s HIV status violates privacy and can result in discrimination, rejection, and violence (52, 53).

### 4.2. Comparison of study findings with previous research

Internalised stigma was experienced as feelings of shame for being infected. Participant narratives revealed the manifestation of internalised stigma as feelings of self-hatred, hopelessness, and worthlessness resulting in self-isolation, exclusion, and several accounts of participants living with a death wish waiting to actualise their suicidal thoughts. The internalisation of stigma was mostly among PLWHA who had low self-esteem and found it difficult to accept their HIV-positive diagnosis resulting in ART non-adherence. Internalised stigma was prevalent among PLWHA who lacked a support system that accepted their HIV-positive status. The internalisation of stigma has also been shown to affect learner’s concentration at school, thus impeding their learning experience (32).

In the present study, participant narratives clearly describe the contribution of internalised HIV-related stigma to mental health issues, notably low self-esteem, self-reported diagnoses of mood disturbances, intrusive thoughts and anxiety, and depression marked by constant suicidal ideation, which they framed as problematic and is consistent with other studies among adolescents (28, 54, 55) and adults (43, 44) conducted in both resource-rich (56, 57) and resource-poor settings (58, 59). In addition, internalised stigma has resulted in anxiety among young PLWHA, jeopardizing their intimate relationships and resulting in diminished sexual or marital prospects consistent with another study in SSA (35). Our findings support previous research findings that PLWHA were worried about spreading their infection to others (60). Despite self-reported mental distress due to HIV-related stigma, most participants did not engage with the mental health system. Only two participants mentioned being a part of support groups that helped them cope with the effects of stigma on their mental health due to the fear of inadvertent disclosure of their status through attendance of support group activities. According to meta-analytic evidence, internalised stigma is associated with negative mental health factors such as increased depressive symptoms, increased psychological distress, lower self-esteem, and lower well-being (61). Participants found it difficult to disclose their serostatus due to the internalisation of stigma. In our setting, not disclosing participants’ HIV status to family, friends, neighbors, and others in the community is regarded as an important protective strategy against HIV-related stigma. HIV-related stigma was identified as a barrier to HIV testing and treatment, as has been noted elsewhere (62, 63). Similarly, our findings match previous work that internalised stigma limits social networks and reduces the social interaction of PLWHA due to self-imposed social isolation, self-exclusion, and the avoidance of negative judgment, guilt, and shame associated with HIV infection (64–66).

Anticipated HIV-related stigma has been theorized to predict medication non-adherence (24, 67) and HIV status non-disclosure (68). In addition, medication adherence has been shown to play a crucial role in managing chronic diseases (69). In the context of HIV, adherence to care and treatment recommendations is essential for ensuring the health, longevity, and a suppressed viral load among PLWHA (70). Our findings support a relationship between anticipated HIV-related stigma and medication non-adherence. Study participants discussed their use of concealment strategies such as avoiding taking medication in public to pass as healthy or normal, purposely refraining from visiting the clinic for drug refills to avoid being seen at the clinic, employing status non-disclosure as an information management strategy, hiding medication and furtively taking medication to avoid inadvertent disclosure. The use of HIV status non-disclosure or concealment in our study is similar to a previous study in a resource limited setting (16). These strategies highlight the lengths individuals went to in order to navigate the challenges posed by stigma and its potential consequences. This suggests a potential link between non-adherence to medication or clinic attendance and anticipated HIV-related stigma, contributing to treatment interruptions as participants report not taking drugs at the appointed times or avoiding care consistent with other studies in SSA (71, 72) and elsewhere (73).

Similarly, our findings are congruent with previous research that the link between medication non-adherence and anticipated HIV-related stigma may result from PLWHA living with fear or anxiety and worrying that medication adherence will lead to inadvertent disclosure of their HIV status (13, 74). HIV status disclosure is regarded as an important predictor of ART adherence (75, 76). From our findings, the fear of anticipated stigma following HIV status disclosure leads to poor or sub-optimal ART adherence, consistent with other studies’ findings (73). In addition, similar to other studies in our context, the fear of damaging parents’ emotional connection with their children and the anticipation of stigma influenced parental readiness to disclose to their children and other community members (14).

In these narratives, perceived stigma significantly impacted PLWHA’s social interactions. Participants spoke of strained relationships with children due to their inability to meet all basic needs as they did before their seropositive diagnosis. Our findings supported a link between perceived stigma and mental health challenges. One participant mentioned external pressure to marry, but he perceived his HIV-positive status as a barrier to marriage. He had only disclosed his status to his mother because he considered it a secret. The relationship between perceived stigma, and status non-disclosure has been reported elsewhere (77). Moreover, this finding supports other studies that have found a link between mental health and perceived HIV stigma (33, 78). Additionally, most participants expressed their mental health challenges using idioms like “thinking too much,” “dislike you,” “become sad,” and “hate you,” which can be equated to the local understanding of mental health challenges. Most participants’ discussion of perceived stigma involved fears of isolation and discrimination closely tied to fear of losing social and material support like food, friendships, and employment, as reported by other studies (62). Participants reported that perceived stigma of PLWHA led to a reluctance to disclose HIV status and poor adherence to ART resulting in physical, social, and emotional isolation as well as disruption in everyday social interactions. According to our findings, perceived HIV stigma led to limited social interactions as participants reported being left out of what other people were planning and children being warned against interacting with peers living with HIV, resulting in physical, social, and emotional isolation. This suggests a possible link between perceived HIV stigma and fewer social interactions, which is consistent with the findings of another study (62).

### 4.3. Study limitations

This qualitative study aimed to provide a contextualized, detailed and rich account of HIV-related stigma experiences and its effects on ART among adults. However, the current study’s findings should be interpreted in light of some limitations. First, because the study was qualitative, we cannot generalize its findings to other resource-constrained settings because data were collected from a single geographical area, and the experiences of HIV-related stigma and its effects are based on the lived experiences of a small number of participants from a specific context. Second, because the study participants were a convenience sample of HIV-positive adults attending an HIV care and treatment clinic, their perspectives may not be representative of the diverse range of experiences of PLWHA in other settings or populations. Third, by using a semi-structured interview guide, we may have missed discussions on other important issues of concern to adults living with HIV. Finally, we only recruited and interviewed adults who were enrolled in care. Findings may not be generalizable to adults living with HIV but not in structured care systems.

### 4.4. Conclusion

Despite the high awareness levels of HIV and AIDS and remarkable increases in ART provision and access in Kenya, adults living with HIV in our setting continue to face HIV-related stigma and discriminatory practices (including self-stigma), albeit to a lesser extent than in the pre-ART era. Participant insights prove that HIV-related stigma negatively influences personal, social, and treatment-related behaviours resulting in deleterious consequences on their physical and mental health. Our findings highlight the urgent need to review, adopt and implement HIV-related anti-stigma campaigns to reduce both individual and community-level forms of stigma. Addressing individual-level stigma will require the design of targeted interventions. The effects of HIV-related stigma, especially on HIV treatment, need to be addressed to improve the lives of adults living with HIV in Kilifi.

## Data availability statement

The raw data supporting the conclusions of this article will be made available by the authors, without undue reservation.

## Ethics statement

The studies involving human participants were reviewed and approved by Scientific and Ethics Review Unit (SERU; Ref KEMRI/SERU/CGMR-C/108/3594), Kenya Medical Research Institute. The patients/participants provided their written informed consent to participate in this study.

## Author contributions

SWW, MKN, and AA conceptualized and gave input to the study design. AA was involved in funding acquisition. SWW and MN supervised data collection. SWW, MKN, and RM collected and curated the data. SWW and AA did project administration and formal data analysis. SWW, MKN, SL, and AA interpreted the data. SWW wrote the first draft of the manuscript. All authors contributed to the article and approved the submitted version.

## Funding

This work was supported by funding from the Medical Research Council (grant number MR/M025454/1) to AA. This award is jointly funded by the UK Medical Research Council (MRC) and the UK Department for International Development (DFID) under the MRC/DFID concordant agreement. It is also part of the EDCTP2 program supported by the European Union.

## Conflict of interest

The authors declare that the research was conducted in the absence of any commercial or financial relationships that could be construed as a potential conflict of interest.

## Publisher’s note

All claims expressed in this article are solely those of the authors and do not necessarily represent those of their affiliated organizations, or those of the publisher, the editors and the reviewers. Any product that may be evaluated in this article, or claim that may be made by its manufacturer, is not guaranteed or endorsed by the publisher.
