# Supplementary material for: A qualitative inquiry of experiences of HIV-related stigma and its effects among people living with HIV on treatment in rural Kilifi, Kenya
Source: Front Public Health. 2023 Jun 21;11:1188446. doi: 10.3389/fpubh.2023.1188446 (PMC10324964; doi:10.3389/fpubh.2023.1188446)
Supplement: Supplementary file 1 [file Data_Sheet_1.pdf]

frontiers

Supplementary Material

A Qualitative Inquiry of Experiences of HIV-related Stigma and its Effects among People Living with HIV on Treatment in rural Kilifi, Kenya

Stanley W. Wanjala[1, 2*], Moses K. Nyongesa[3, 4, 5], Rachael Mapenzi[3], Stanley Luchters[1, 6, 7], Amina Abubakar[3, 5, 8, 9*]

*** Correspondence:** Stanley W. Wanjala; Amina Abubakar
s.wanjala@pu.ac.ke; amina.abubakar@aku.edu

Supplemental Table 1: Interview Guide for in-depth interviews with Adults living with HIV in Kilifi

**Instructions to the interviewer:**<br>Before the interview begins, conduct the informed consent.<br>Also, collect demographic data from the interviewee (sex, age, education level, religion, place of residence) and record in the register.
Introduction (5 m)<br>- Welcome participants and introduce yourself.<br>- Explain the general purpose of the discussion and why the participant was chosen.<br>- Discuss the purpose and process of the in-depth interview<br>- Explain the presence and purpose of recording equipment<br>- Address the issue of confidentiality, e.g., that information discussed will be analyzed as a whole and that the participants' names will not be used in any analysis of the discussion.
**Discussion Guidelines**
**1. Challenges of living with HIV**<br>There are specific challenges that people living with various chronic conditions (cancer, hypertension, HIV, etc.) face.<br>In your opinion, what challenges do adults living with HIV/AIDS face in their day to day life? Be it at WORK or in the COMMUNITY?<br>Probe ...

- Acceptance of HIV status<br>- Stigma and discrimination (if raised see below)<br>- Adherence to ARVs<br>- Disclosure difficulties (if raised, ask…to whom and why it is easy/difficult to disclose or not disclose)
**2. Stigma and discrimination**<br>I would like us to talk more about stigma and discrimination against adults living with HIV (if mentioned earlier, IF NOT, introduce the topic…<br>♣ What are the indicators of stigma/discrimination against adults living with HIV?<br>♣ In your opinion, can stigma and discrimination against an adult living with HIV affect them in any way?<br>Probe about:<br>○ Effect of stigma and discrimination on disclosure<br>○ Effect of stigma and discrimination on ARV adherence<br>○ Effect of stigma and discrimination on Mental health<br>○ Effect of stigma and discrimination on relationships/socialization<br>We have reached the end. Is there anything else that we have not discussed that you think is important to talk about?

Supplemental Table 2: Research Team Attributes and Qualifications

**Author**	Attributes and Qualifications
S.W.W.	Male social scientist with a Master of Arts in Medical Sociology, a Bachelor of Arts degree in Sociology, qualitative research methods training, and global health training. His research interests include HIV-related stigma as well as maternal and child health.
M.K.N.	A male trained nurse with a Doctorate degree in Global Mental Health, a Master's in Global Mental Health, Diploma in Health Research Methods, and qualitative research training. He is interested in disease comorbidity research intersecting between psychiatric disorders and HIV/AIDS and the testing of psychological interventions seeking to address mental disorders comorbid with HIV using m-health platforms.
R.M.	Female field worker at the Kenya Medical Research Institute/Wellcome Trust Research Programme, Centre for Geographic Medicine Research (Coast), Kilifi, Kenya. She is a team member in the neuro assessment group. She is responsible for identifying and recruiting study participants and data collection.
S.L.	Male professor with a medical degree, an MSc in Public Health for Developing Countries, and a Ph.D. in Health Sciences. His work has centered on sexual and reproductive health and maternal and child health. Within this field, he has advanced several focus areas, including the Involvement of men in improving maternal and neonatal health outcomes, the effects of climate change on maternal and child health, Sexually transmitted infections, and key populations such as sex workers and people who inject drugs.
A.A	A female professor who is a Developmental Psychologist with a Ph.D. in the neurodevelopmental assessment of children in rural sub-Saharan Africa. Her thematic focus is on children at risk of experiencing developmental delays due to exposure to various health problems, particularly HIV, malnutrition, and malaria. Her research interests include quantifying the neurocognitive burden of early childhood diseases, developing culturally appropriate psychological measures for use in SSA, and identifying culturally appropriate intervention strategies for at-risk children in SSA.

Supplemental Table 3. CORE-Q (Consolidated criteria for Reporting Qualitative research) Checklist

Item No.	Topic	Guide Questions/Description	Reported on Page No.
**Domain 1: Research team and reflexivity**			
Personal Characteristics			
1	Interviewer/facilitator	Which author/s conducted the interview or focus group? *Data from in-depth interviews are presented in this manuscript.*	Page 4, Supplemental Table 2
2	Credentials	What were the researcher's credentials? *The manuscript presents interviewer-facilitated data; thus, we describe the credentials of the researchers who participated in this study.*	Page 4, Supplemental Table 2
3	Occupation	What was their occupation at the time of the study? *We describe the occupations of the researchers on the study team at the time of the study.*	Page 4, Supplemental Table 2
4	Gender	Was the researcher male or female? *The genders and initials of all the researchers who participated in each stage of the study team are described for our readers to make a connection between the individuals and names within the authorship.*	Pages 4, Supplemental Table 2
5	Experience and training	What experience or training did the researcher have? *We describe both the experience and training of each researcher on the study team.*	Supplemental Table 2
Relationship with participants			
6	Relationship established	Was a relationship established prior to study commencement? *Researchers elucidated the study objectives to potential participants and informed them of their right to decline participation or withdraw consent at any research stage. All participants provided written, informed consent to be part of the study.*	Page 5
7	Participant knowledge of the interviewer	What did the participants know about the researcher? (e.g., personal goals, reasons for doing the research) *Researchers engaged with participants with respect to audio-recording of HIV-related stigma conversations. Potential participants were informed about the study objectives and their right to decline participation or withdraw consent at any research stage.*	Page 5
8	Interviewer characteristics	What characteristics were reported about the interviewer/facilitator? e.g., bias, assumptions, reasons and interest in the research topic? *Potential participants were acquainted with the study objectives and their right*	Page 5

		to decline participation in the study or withdraw consent at any research stage without any consequence.	
**Domain 2: Study design**			
Theoretical framework			
9	Methodological orientation and theory	What methodological orientation was stated to underpin the study? e.g., grounded theory, discourse analysis, ethnography, phenomenology, content analysis. *We employed the framework analysis in this study.*	Pages 4
Participant selection			
10	Sampling	How were participants selected? e.g., purposive, convenience, consecutive, snowball. *We used a convenience sample selected study participants depending on availability and willingness to participate.*	Page 4
11	Method of approach	How were participants approached? e.g., face-to-face, telephone, mail, email. *Participants were mainly recruited via a phone call by a research team member after they were identified using records from past quantitative studies conducted at the health facility.*	Page 4
12	Sample size	How many participants were in the study? *There was a total of 40 qualitative respondents.*	Page 5, Table 1
13	Non-participation	How many people refused to participate or dropped out? Reasons? *Participation in the in-depth interviews was voluntary and data related to refusal to participate or drop out is unknown.*	n/a
Setting			
14	Setting of data collection	Where was the data collected? e.g., home, clinic, workplace. *Participants were mainly recruited via a phone call and were interviewed in a clinic space.*	Page 4
15	Presence of non-participants	Was anyone else present besides the participants and researchers? *No one else was present besides the researcher and participants.*	Page 4
16	Description of sample	What are the important characteristics of the sample? e.g., demographic data, date. *Participants' socio-demographic data are described on Page 5 and presented in Table 1.*	Page 5, Table 1
Data collection			
17	Interview guide	Were questions, prompts, guides provided by the authors? Was it pilot tested? *The interview guide and probes are provided.*	Supplemental Table 1
18	Repeat interviews	Were repeat interviews carried out? If yes, how many? *There were no repeat interviews carried out in this study.*	n/a
19	Audio/visual recording	Did the research use audio or visual recording to collect the data? *Recorders were used to audio-record interviews, which were later transcribed verbatim, translated into English, reviewed for accuracy, and uploaded on Nvivo*	Page 4

20	Field notes	Were field notes made during and/or after the interview or focus group? *n/a*	n/a
21	Duration	What was the duration of the interview or focus group? *The interviews lasted between 30-45 minutes*	Page 4
22	Data saturation	Was data saturation discussed? *The emergence of data saturation which was used to determine the sample size during the data collection exercise was discussed by the researchers*	Page 4
23	Transcripts returned	Were transcripts returned to participants for comment and/or correction? *n/a*	n/a
Data analysis			
24	Number of data coders	How many data coders coded the data? *We provide information describing the number, role and identification of all data coders for this study.*	Page 4, Supplemental Table 2
25	Description of the coding tree	Did authors provide a description of the coding tree or codebook? *no*	
26	Derivation of themes	Were themes identified in advance or derived from the data? *Themes were inductively derived from raw data and deductively by drawing on questions from the interview guide.*	Page 4
27	Software	What software, if applicable, was used to manage data? *We used NVIVO 11 software.*	Page 4
28	Participant checking	Did participants provide feedback on the findings? *We did not consent patients/caregivers to provide feedback on findings.*	n/a
Reporting			
29	Quotations presented	Were participant quotations presented to illustrate the themes/findings? Was each quotation identified? e.g., participant number. *Representative quotes are embedded within the text*	Pages 5-12
30	Data and findings consistent	Was there consistency between the data presented and the findings? *We demonstrate consistency between the data presented in the Results section and the interpretation of findings in the Discussion section.*	Pages 5-12
31	Clarity of major themes	Were major themes clearly presented in the findings? *We presented all major themes in detail.*	Pages 5-12, Figure 1
32	Clarity of minor themes	Is there a description of diverse cases or discussion of minor themes? *We provide a variety of rich quotes within the text.*	Pages 5-12

Adapted from: Tong A, Sainsbury P, Craig J. Consolidated criteria for reporting qualitative research (COREQ): a 32-item checklist for interviews and focus groups. *International Journal for Quality in Health Care*. 2007. Volume 19, Number 6: pp. 349 – 357.https://doi.org/10.1093/intqhc/mzm042
